# Oculocardiac reflex during endoscopic transsphenoidal removal of pituitary adenoma

**DOI:** 10.4103/0019-5049.65361

**Published:** 2010

**Authors:** Tumul Choudhury, Hemanshu Prabhakar, Gyaninder Pal Singh, Ashish Bindra

**Affiliations:** Department of Neuroanesthesiology, All India Institute of Medical Sciences, New Delhi, India

**Figure 1:**
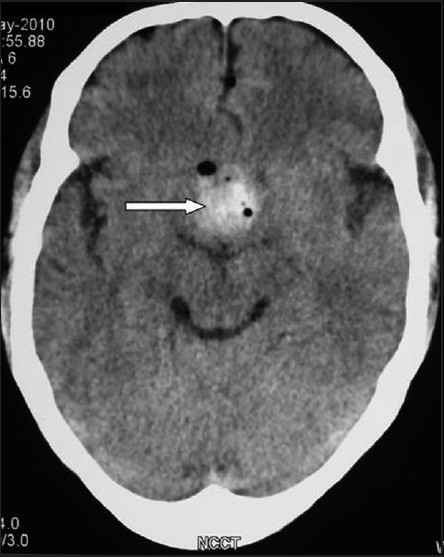
A computed tomographic scan of the head showing a large haematoma in the sellar region (bold white arrow)

Sir,

With the advent of endoscopic neurosurgery, the endoscopes have now been applied to access intracranial tumours with favourable result. The literature revealed that this method has very few complications, including vision loss.[1] We report a case of sudden bilateral vision loss after endoscopic surgery for pituitary tumour, where intraoperative haemodynamic instability was possibly suggestive of a devastating postoperative complication. A 23-year-old, 70-kg male presented to the neurosurgical department with a complaint of coarsening of features for past 2 years. He was a known case of diabetes mellitus. A clinical diagnosis of acromegaly was confirmed by his growth hormone (GH) assessment (GH >90). Other investigations were within normal limits. Magnetic resonance imaging (MRI) revealed a 2 × 2 × 1.8 cm suprasellar lobulated mass, suggestive of pituitary tumour. His preoperative vision was 6/6 in both eyes. The patient was scheduled for endoscopic transsphenoidal removal of pituitary macroadenoma. Anaesthesia was induced with fentanyl 2 mcg/kg, propofol 2 mg/kg and rocuronium 1 mg/kg. Standard monitoring was applied. Anaesthesia was maintained with propofol infusion and a oxygen–nitrous oxide mixture (1:2) along with intermittent boluses of fentanyl and rocuronium. At the time of tumour removal, sudden bradycardia occurred and the heart rate dropped from 86 beats per minute (bpm) to 37 bpm. The surgeon was immediately informed, who stopped further manipulation and within 15 s, the heart rate came back to the baseline value. During these episodes, the mean blood pressure remained near baseline value of 85 mmHg and did not fall significantly. The surgery was allowed to continue and there occurred three episodes of bradycardia. All these episodes occurred when surgeon was operating in close proximity to the optic chiasma. No pharmacological intervention was required during any of the episode of bradycardia. At the end of surgery, anaesthesia was discontinued and neuromuscular block reversed with neostigmine 0.5 mg/kg and glycopyrrolate 10 mcg/kg. The patient was shifted to the neurosurgical intensive care unit (ICU). Six hours later, the patient complained of bilateral vision loss. Ophthalmic examination revealed bilateral negative perception of light. Immediately, computed tomography was performed which revealed haematoma of 10 mm × 7 mm size in the sellar region, compressing optic chiasma, along with remnants of the tumour [Figure 1]. Immediately craniotomy and evacuation of haematoma was performed. Postoperatively, his ophthalmic examination revealed a 6/6 vision in the right eye while in the left eye only finger counting was present. No further improvement in vision of the patient was observed during his hospital stay. The mechanisms involved in visual complications include direct injury or devascularisation of the optic apparatus, fracture of the orbit, postoperative haematoma, cerebral vasospasm and prolapse of the optic chiasm into an empty sella[1] and tension pneumosella.[2] During the postoperative period, our patient developed bilateral vision loss that may be due to tumour residue and optical chiasmal compression by sellar haematoma. However, intraoperative events of repeat bradycardia, possibly resulting from the oculocardiac reflex cannot be ignored. The reflex elicited may act as a warning signal and at the same time predict the surgical outcome also.
